# Inertial properties of the German Shepherd Dog

**DOI:** 10.1371/journal.pone.0206037

**Published:** 2018-10-19

**Authors:** O. Yvette Jones, Silvia U. Raschke, Philip E. Riches

**Affiliations:** 1 Centre for Applied Research and Innovation, British Columbia Institute of Technology, Burnaby, British Columbia, Canada; 2 Department of Biomedical Engineering, University of Strathclyde, Glasgow, United Kingdom; University of Bari, ITALY

## Abstract

One of the most popular dog breeds deployed by both the police and military has been the German Shepherd yet little is known about the morphology or body segment parameters of this breed. Such measures are essential for developing biomechanical models which, in turn, may guide clinicians in developing surgical interventions, injury treatment and prevention procedures. This paper provides a complete set of body segment parameters and inertial properties for the German Shepherd. Morphometric measures and 3-dimensional inertial properties, including mass, centre of mass, moment of inertia and volume, were measured from 17 segments from 6 German Shepherd police service dog cadavers. Using whole body mass and geometric modelling, 11 regression equations were developed for predicting segment masses, and 33 equations were developed for predicting moments of inertia. Using these data, inverse dynamic analyses may be applied in future investigations of canine mechanics, guiding surgical procedures, rehabilitation and training especially for the German Shepherd breed but potentially for other breeds too.

## Introduction

Before 1890 police dogs had no formal training and were used primarily as trackers or as guard dogs. However, by 1895 both Belgium and France successfully began training dogs to work with police on foot patrol. By the First World War the breed was serving as medical service dogs, sentinels, trackers, messengers, supply carriers and guard dogs [1, 2]. Today, the German Shepherd is arguably the most popular breed of dog deployed in police work and in the military with 112 teams within the Royal Canadian Mounted Police (RCMP) alone.

Considerable expense is placed attaining and maintaining police dog teams, and they become significantly more costly if a dog becomes injured, not only due to veterinary fees but the department also loses a canine team until the dog is back in service. Knowledge of canine veterinary musculoskeletal disease and surgical techniques has seen significant developments however evaluation of functional outcomes are still primarily subjective and based on the experience and observational skills of the clinician. As with humans and horses, certain conditions will present with different biomechanical signatures, opening up the possibility that an objective clinical gait evaluation would be a useful diagnostic tool for characterizing gait [3–5]. Dynamic analysis of limb movements requires the input of morphometric data, commonly referred to as body segment parameters (BSPs), which typically include segment dimensions, mass, centre of mass (CoM), inertial tensors and segment density or volume. Such data are essential for input into a linked segment model so that forces and moments specific to each joint may be obtained.

The importance of BSPs to the study of human motion and ergonomics has been known since 1681 [6]. Direct measurements of sample populations can be taken via cadaver segmentation [7–9] or from living subjects through the use of scanning methods (γ-ray, CT, MRI) [10] and geometrical modelling based on anatomical measures [11]. The greatest benefit to using cadaver segmentation for determining BSPs is that it permits direct measurements of an individual segment’s mass, volume, CoM, and moments of inertia (MoI), quantities that express an object’s resistance to angular acceleration about three orthogonal axes. Therefore, this method should be considered “gold standard” in comparison to other less direct methods which involve assumptions pertaining to tissue density and segment geometry.

Many kinematic [4, 12–14] and kinetic [15–17] studies have described gait characteristics of dogs, with some including inverse dynamics to determine joint force, torques and powers [18–20] however these have not included the contribution of segment inertial properties [21]. Editorials on canine research state that there is a lack of body segment parameter data [22] and a “…need to develop a database of accurate limb segment morphometric descriptions for specific (dog) breeds, and accumulate a database of normal, breed-specific joint kinetics….” [23]. Full and partial equine BSP data exist [24–28], whilst canine 2D BSP data have been determined from cadavers from mixed-breed dogs [29, 30] and BSP data exist for the hind limb of Labrador Retrievers [31]. The only complete set of 3D BSP data have been obtained through MRI on three mixed breed dogs separated into 15 segments [32]. To enable extrapolation to other dogs, the CoM was normalised to the length of the segment and MoI normalised by the square of the segment length [32–34]. No full 3D data set exists for the dog based on cadaveric segmentation and, despite the importance of the German Shepherd in the emergency and public services of many countries, no specific data exist for this breed, with the exception of the skull, e.g. [35].

It is hypothesized that body segment parameters and 3-dimensional inertial properties for the dog may be directly measured from cadaveric specimens. This study aims to measure and record segment morphometry, masses, CoMs, inertial tensors and volumes of German Shepherd dogs. Based on these data, regression equations are determined for the estimation of segment inertial properties for other German Shepherds and potentially other large dog breeds.

## Materials and methods

The RCMP uses only purebred German Shepherds for their general duty teams. On rare occasion a dog dies suddenly due to unforeseen conditions or illness, or must be euthanized due to an accident, medical or behavioural issues. The RCMP supported this research by donating these dogs so that they may one day assist in promoting the health and rehabilitation of future dogs. Upon getting word that a dog had become available, the researchers would collect the dog from the attending veterinarian. Written informed consent was obtained: participants were provided with full disclosure of the study, including; the purpose, data collection methods and confidentiality procedures and were informed that they were free to withdraw from the study at any time. The study team, in 2006 at the outset of the study, approached the Canadian Council on Animal Care and the University of British Columbia Animal Care Committee and asked them to review the study. Both granted a waiver, in accordance with ethical guidelines at the time, their reason being that animals were recruited post mortem and no animal was specifically euthanised for the study.

### Dogs

Morphometry and inertial measurements were collected from five purebred German Shepherds from January 2007 to March 2009, with a sixth dog added in February 2014. The dogs were required to be at least 2 years of age and, to ascertain muscle mass, to either be on active duty at time of death or retired but still active, as reported by their handler. Exclusion criteria included: dogs exhibiting congenital or traumatic abnormalities, structural atrophy, excessive weight loss or obesity. Of seven potential dogs, six male dogs fulfilled these criteria, with one dog declined inclusion due to significant physical injury. The dogs had with a mean age of 4.75 years (range 2 to 8.5 years) and a mean body mass of 36.8 kg (range 34.29 to 39.41 kg).

### Preliminary measures and freezing

Similar to previous research [36–38], morphometric dimensions were recorded and selected landmarks identified and marked on the skin prior to freezing. The dogs were not shaved so as not to affect segment mass. Dogs were placed in a neutral stance position via a custom harness and frame. Since no standard anatomical neutral position has been established for the dog, the selection of “neutral position” was loosely based on the relaxed four-footed stance with tail down, mouth closed and right and left sides placed symmetrical. Dogs were frozen at -18°C for a minimum of 48 hours to extend the handling time and to form a rigid body for subsequent measurements.

### Segmentation and segment definitions

**Fig 1:**
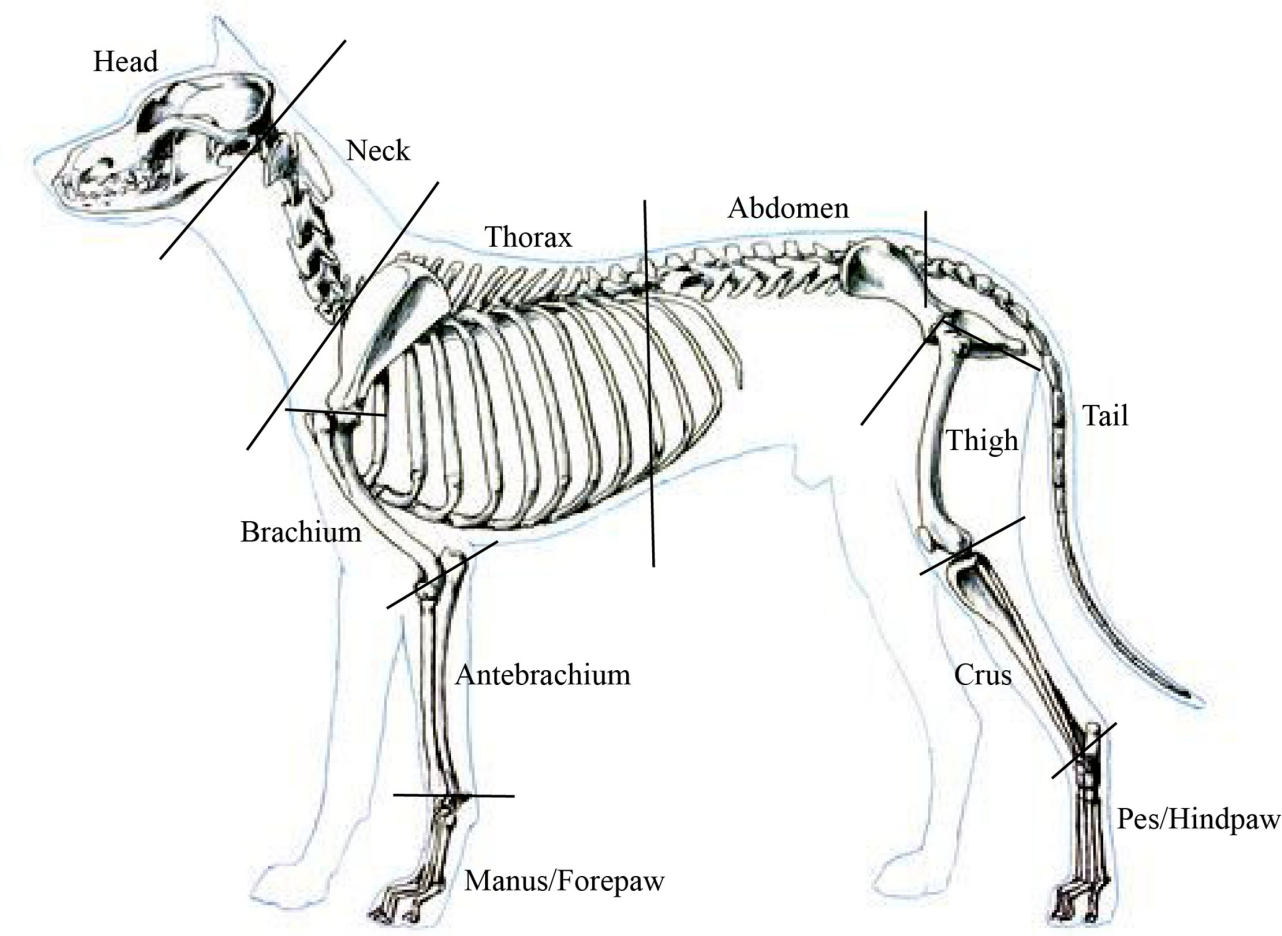
Seventeen-segment model of the dog showing segmentation lines.

Each frozen cadaver was divided into 17 segments (Fig 1). All cuts passed through the estimated instantaneous joint centre of rotation, similarly identified and demonstrated by for humans [7, 9], for horses [26, 28] and for dogs [39, 40]. Cuts were made using a battery powered reciprocating saw, a hand saw and an electric cast cutter (Stryker 9002–210). Lost tissue was weighed for inclusion in the summation of segment masses. Upon dissection, segments were weighed, photographed, and placed in tightly sealed plastic freezer bags to control dehydration and returned to the freezer.

#### Head/neck

With the head at approximately 125 degrees to the cervical spine, separation of the head from the neck passed along a plane tangent to the midpoint between the external occipital protuberance and the cranial point of the spinous process of the axis (keeping the ears intact) and the right and left paracondylar processes.

#### Neck/thorax

With the cervical spine at approximately 150 degrees to the thoracic spine, separation of the neck from the thorax passed along a plane tangent to the greater tubercle of the humerus and the cranial border of the scapula on the right and left sides.

#### Brachium/thorax

Due to the difficulty of achieving a clean separation, the scapula was included as part of the thorax. Separation of the brachium from the thorax required a compound cut; the first was on the underside of the brachium following the axial crease upward along the thorax to the humeral head, the second ran across from the greater tubercle of the humerus, tangent to the midline between the acromion and humeral head to the apex of the axial crease.

#### Brachium/antebrachium

The brachium was separated from the antebrachium along a plane passing through the medial and lateral humeral epicondyles and a point at the crease of the elbow. Note that, in stance, the flexure surface of the brachium is approximately 145 degrees relative to the antebrachium, causing the olecranon tuber to be partially bisected within the brachial segment.

#### Antebrachium/manus

The antebrachium was separated from the manus along a plane tangent to the radial and ulnar styloid processes and the accessory carpal bone, keeping it intact. The cranial surface of the antebrachium is approximately 170 degrees relative to the manus.

#### Thorax/abdomen

On observation, the canine thorax can rotate separately from the abdomen, particularly if changing direction while running however there are few studies documenting the specific movement of the thoracic spine in relation to the lumbar spine [41–43]. It was decided that the thorax and abdomen would be measured separately in order to provide an opportunity for future investigation in this area. The plane of separation of the thorax from the abdomen was made tangent to the midpoint between T13 and L1 spinous processes and the midpoint between the xyphoid process and the umbilicus.

#### Abdomen/thigh

The separation of the thigh from the abdomen required a compound cut. The caudal plane passed from the point of the ischial tuberosity to the femoral head at the ventral edge of the acetabulum. The cranial plane passed diagonally from the apex of the thigh crease to the most dorsal projection of the greater trochanter, through the femoral head to the acetabulum to meet the first cut.

#### Thigh/crus

Similar to brachium/antebrachium, the separation of the crus from the thigh passed along a plane bisecting through the femoral condyles at the proximal attachments of the collateral ligaments and a point at the crease at the back of the stifle joint with the stifle flexed at approximately 100 degrees.

#### Crus/pes

Separation of the pes from the crus was made with the pes flexed at 95 degrees to the crus. The cut plane passed just distal to the lateral and medial malleoli and tangent to the dorso-caudal edge of the calcaneus.

#### Abdomen/tail

The tail was removed along a plane separating the sacrum from the first caudal vertebra.

### Experimental procedures

#### Mass

Whole body mass was measured prior to dissection using a Bertec 4060–10 strain-gauge-based force plate (Bertec Corporation, Columbus, OH, USA). Segment mass measures were completed directly using a Mettler Dual Range Precision Balance. The balance has a capacity of 32100g and is accurate to 0.1g for measures below 6400g, 1.0g for measures over 6400g. (Mettler Toledo Canada, Mississauga, ON, CA). To monitor any loss of mass due to sublimation [44], segment masses were taken at three separate instances: immediately upon dissection, again approximately 1–2 days later at the same time inertial properties were taken, and a third time approximately 1 week later when volumetric measures were completed. An average of the 3 measures was taken for use in inertial calculations.

#### 3-D coordinate system

**Fig 2:**
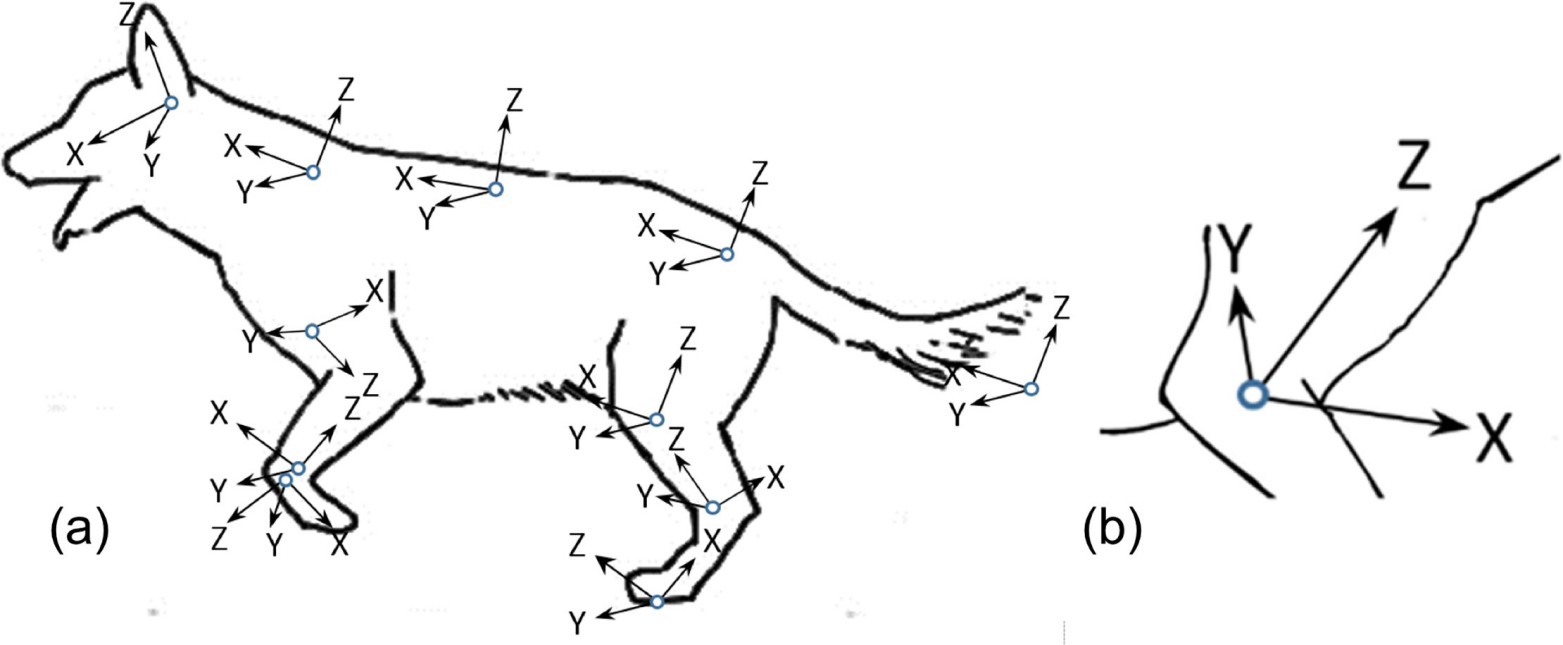
Segment axis orientation. (a) Segment axis orientation established by the segment holder; (b) right brachium axes.

The irregular shape of the segments prompted the creation of a segment holder similar to those used in previous studies [8, 36]. The boxes were constructed of Styrofoam, affording thermal isolation, and optimally sized to fit each segment as closely as possible. Each of the boxes were marked with six axes: xx, yy, zz, xy, xz and yz axes where xy, xz and yz are axes coplanar but nonparallel to xx, yy and zz (for full detail see [8]). Using foam saddles and tape, each frozen segment was securely positioned in the box such that box xx, yy and zz axes defined a segment’s coordinate system (Fig 2A), with the yy axis for all segments corresponding to the flexion/extension axis and oriented right to left for all segments except the crus. Note that for ease of measurement procedures during the experiment it was necessary to reverse the orientation of the right brachium hence its axes are also reversed (Fig 2B).

#### Measures

MoI for each segment was measured using the simple pendulum method [36]. Using strings attached to connectors on the box wall, the each box was suspended from a rigid A-frame; the abdomen and thorax were suspended directly using 27 kg rated stainless steel wire. The segment holder was swung in the vertical plane and the period of oscillation, length of pendulum and mass of the segment were recorded. A Vicon MX motion capture system (Vicon, Oxford, UK) recorded the period of oscillation for the box and the mean taken over 3 successive trials. MoIs were measured about the 6 box axes with and without the segment in situ. The MoIs about the xx, yy and zz axes provided the MoIs of each segment around the axes identified in Fig 2. Subtracting the known mechanical properties of each empty box (mass, CoM, MoI) from the composite (box plus segment), the MoI for each segment about its CoM was then calculated as follows:

$$l_{CoM}=\frac{{mg}_{c}l_{c}T_{c}^{2}-{mg}_{b}l_{b}T_{b}^{2}}{4\pi^{2}}-m_{s}l_{s}^{2}$$

Where the subscript *c* refers to the composite box, subscript *b* refers to the empty box and subscript *s* refers to the segment.

Product MoI were calculated as follows [8, 24, 28]:

$$I_{xy}=\frac{I_{yy}+I_{xx}{tan}^{2}\theta -(l+{tan}^{2}\theta )I_{xy}^{*}}{2tan\theta }$$

where $I_{xy}^{*}$ is the MoI about the box’s xy axis; *l* the length of the pendulum; and *θ* is the angle formed by the intersection of the yy and xy axes. *I*_*xz*_ and *I*_*yz*_ are calculated in a similar manner. CoMs were determined by balancing the segment box on a knife edge in the three axes.

A total of 100 morphometric dimensions were gathered from each dog: total body mass, 23 separate measures for the forelimbs, 33 for the hind limbs, and 43 for the rest of the body. To assess repeatability, multiple sets of measures were conducted on select dimensions prior to segmentation and approximately one week later after segmentation. Paired-sample t-tests were conducted to confirm no significant differences existed between measurement sets taken at the different times of the study. Significant differences (p < .05) between a compared pair resulted in the measure being excluded from the model.

#### Regression analyses

Regression equations were created from whole body mass and geometric modelling using morphometric dimensions to predict segment masses and MoIs. Different morphometric dimensions were used in a variety of non-linear regression equations, based on different assumed segment geometric shapes, to determine which function best correlated to the segment parameter. The geometries tested included: a cylinder, a rectangular pyramid, a cone, a conical frustum, and an ellipsoid. Left and right sides were combined to provide n = 12 for this analysis.

## Results

### Morphometry and body segment parameters

At the start of the research it was unknown which data would provide the best regression correlations therefore a variety of dimensions were evaluated based on currently used human models, animal studies as well as on geometric shapes. Segment data were averaged across all dogs: Table 1 summarizes segment masses (normalized to body mass), CoMs, densities and volumes; Table 2 presents the calculated MoI. There was no loss of segment mass over storage time due to sublimation and tissue loss due to cut waste was 182g or 0.49% of total body mass per dog. This was considered negligible.

**Table 1:** Normalised segment masses, CoMs, densities and volumes.

Segment	Endpoints (cran-caud or prox-dist)	Segment Mass/ Total Mass			CoM / Segment Length						Density (kg/m^3^)			Volume (cm^3^)		
		mean		SD	R_prox/cran_		SD	R_dist/caud_		SD	mean		SD	mean		SD
Manus	Mid carpus to distal 3rd phalanx	0.0072	±	0.0002	0.4848	±	0.0089	0.5185	±	0.0091	934.02	±	18.10	287	±	12.9
Ante-brachium	Lateral epicondyle to carpal jt	0.0138	±	0.0005	0.3941	±	0.0055	0.6076	±	0.0053	977.54	±	17.10	521	±	30.7
Brachium	Glenohumeral jt to lat epicondyle	0.0240	±	0.0013	0.4183	±	0.0183	0.5869	±	0.0197	974.37	±	12.71	908	±	86.2
Pes	Tuber calcaneus to distal 3rd phalanx	0.0082	±	0.0002	0.5140	±	0.0044	0.4880	±	0.0042	1013.58	±	12.45	305	±	12.6
Crus	Femoral condyle to lateral maleolus	0.0150	±	0.0006	0.3659	±	0.0113	0.6364	±	0.0111	1010.60	±	19.38	548	±	33.3
Thigh	Greater trochanter to femoral condyle	0.0451	±	0.0028	0.4463	±	0.0115	0.5601	±	0.0124	939.78	±	13.40	1760	±	183.0
Head	Inion to prosthion	0.0770	±	0.0039	0.3165	±	0.0081	0.6842	±	0.0082	1004.18	±	20.29	1000	±	20.3
Neck	Atlas/axis to C7/T1	0.0661	±	0.0068	0.5627	±	0.0122	0.4431	±	0.0128	970.01	±	25.53	2550	±	292.0
Abdomen	T13/L1 to tail base	0.2415	±	0.0153	0.4677	±	0.0047	0.5343	±	0.0050	963.89	±	7.71	9260	±	714.0
Thorax	C7/T1 to T13/L1	0.3806	±	0.0101	0.5368	±	0.0110	0.4670	±	0.0109	1083.39	±	83.44	13300	±	1070.0
Tail	Base of tail to tip	0.0080	±	0.0005	0.3128	±	0.0158	0.6892	±	0.0162	900.63	±	63.73	345	±	45.1

**Table 2:** Moments and products of inertia (kg.m^2^).

Segment	I_int/ext_			I_abd/add_			I_flx/ext_			I'_xy_			I'_xz_			I'_yz_		
	mean		SD	mean		SD	mean		SD	mean		SD	mean		SD	mean		SD
Manus	6.37	±	0.79	10.26	±	1.25	9.83	±	1.17	2.64	±	1.11	4.02	±	1.01	1.68	±	0.74
Antebrachium	9.82	±	1.23	32.68	±	2.64	26.86	±	1.85	6.84	±	1.12	24.32	±	4.67	-5.33	±	5.57
Brachium	29.87	±	4.06	51.46	±	8.07	43.69	±	7.16	8.85	±	3.27	17.16	±	2.96	19.12	±	9.80
Pes	18.86	±	1.59	8.93	±	0.80	17.34	±	0.51	5.36	±	0.82	7.29	±	0.76	2.40	±	0.98
Crus	15.48	±	1.95	28.55	±	1.98	28.00	±	1.69	3.77	±	3.18	29.28	±	2.49	3.14	±	6.80
Thigh	100.61	±	9.24	79.74	±	12.24	109.91	±	11.69	19.00	±	3.27	28.01	±	3.71	38.78	±	3.51
Head	253.67	±	5.53	191.61	±	16.20	248.60	±	10.24	51.79	±	5.16	78.18	±	6.94	82.20	±	7.29
Neck	155.04	±	15.89	160.27	±	11.19	142.75	±	19.62	50.00	±	7.23	34.66	±	3.88	46.17	±	9.04
Abdomen	1164.68	±	185.52	737.53	±	105.81	1457.73	±	213.35	162.88	±	20.11	246.50	±	58.04	70.23	±	283.44
Thorax	2811.71	±	160.47	1350.62	±	35.68	2825.10	±	170.99	318.51	±	10.12	701.83	±	43.53	1004.15	±	153.99
Tail	8.21	±	1.41	60.70	±	4.37	48.50	±	4.27	21.19	±	2.41	-435.32	±	1915.34	-2039.36	±	628.28

### Regression correlations

Correlations to geometric shape were applied using a variety of combinations of the dimension. When tested separately, results for the right and left limbs were significant for many of the applied combinations. The number of significant correlations were drastically reduced when the combined right and left MoI data were compared with the models. Multiple regression correlations of body mass and geometric model produced much better results and so it was this approach that was used for the majority of the correlations. Regression equations for predicting segment masses and MoIs, along with their R^2^ values, are given in Tables 3, 4 and 5.

**Table 3:** Regression equations for determining segmental mass and MoIs about 3 primary axes—Forelimb.

Segment	Regression Equation				R^2^	Notes	
Manus	mass	m	=	-0.003a + 69.762bc^2^ + 0.077	0.867	a	Whole body mass (kg)
	internal/external	I_xx_	=	-0.00012a + 0.0052	0.721	b	length of caudal edge of met pad to podactylion III
	flexion/extension	I_yy_	=	-0.00015a + 0.00654	0.689	c	Bistyloidal circumference at carpal joint (m)
	abduction/adduction	I_zz_	=	0.00018a - 0.00558	0.870		
Antebrachium	mass	m	=	0.012a + 101.084bc^2^–0.463	0.903	a	Whole body mass (kg)
	abduction/adduction	I_xx_	=	-0.00032a + 0.00066a(0.076c^2^+b^2^) + 0.01406	0.595	b	length of antebrachium—proximal to distal joint centers (m)
	flexion/extension	I_yy_	=	-0.00005a + 0.00160a(0.076c^2^+b^2^) + 0.00168	0.459	c	Bistyloidal circumference at carpal joint (m)
	internal/external	I_zz_	=	0.0001a + 0.00173ab^2^–0.00496	0.886		
Brachium	mass	m	=	0.092a + 25.062b(c^2^+cd+d^2^) - 3.811	0.646	a	Whole body mass (kg)
	abduction/adduction	I_xx_	=	0.00053a + 0.00223a(c/π)^2^+b^2^–0.1990	0.883	b	length of brachium—proximal to distal joint centers (m)
	flexion/extension	I_yy_	=	0.00082a + 0.00465a(c/π)^2^+b^2^–0.03308	0.728	c	circumference at highest point in the axilla, perpendicular to long axis (m)
	internal/external	I_zz_	=	0.00015a + 0.003a(c^5^-d^5^)/(c^3^-d^3^) - 00.02139	0.408	d	circumference at olecranon process across epicondyles (m)

**Table 4:** Regression equations for determining segmental mass and MoIs about 3 primary axes–Hind limb.

Segment	Regression Equation				R^2^		Notes
Pes	mass	m	=	-0.009a + 633.875bcd + 0.357	0.895	a	Whole body mass (kg)
	abduction/adduction	I_xx_	=	0.00009a - 0.00256	0.870	b	length of proximal joint center to podactylion III (m)
	flexion/extension	I_yy_	=	-0.00007a + 0.00417	0.687	c	breadth across metatarsal phalangeal joints (m)
	internal/external	I_zz_	=	-0.00016a + 0.00786	0.594	d	depth of pes at midpoint between malleoli and base of the pes (m)
Crus	mass	m	=	0.075a + 139.127bc^2^–3.393	0.576	a	Whole body mass (kg)
	abduction/adduction	I_xx_	=	0.00023a + 0.00137a(0.076c^2^+b^2^) - 0.00785	0.903	b	length of crus—proximal to distal joint centers (m)
	flexion/extension	I_yy_	=	0.00021a + 0.00108a(0.076c^2^+b^2^) - 0.00659	0.985	c	circumference of hock at malleoli (m)
	internal/external	I_zz_	=	0.00009a - 0.00423ac^2^ + 0.00454	0.941		
Thigh	mass	m	=	0.018a + 105.849bc^2^–0.711	0.709	a	Whole body mass (kg)
	abduction/adduction	I_xx_	=	-0.00091a + 0.00258a(0.076c^2^+b^2^) + 0.03813	0.732	b	mid centroid of femoral head (m)
	flexion/extension	I_yy_	=	-00.0006a + 0.00536a(0.076c^2^+b^2^) + 0.02502	0.792	c	circumference of stifle joint at mid-patella (m)
	internal/external	I_zz_	=	-0.00067a + 0.00001ac^2^ + 0.03467	0.466		

**Table 5:** Regression equations for determining segmental mass and MoIs about 3 primary axes–Body.

Segment	Regression Equations				R^2^		Notes
Head	mass	m	=	0.243a + 1602.163bc^2^–11.739	0.778	a	Whole body mass (kg)
	internal/external	I_xx_	=	0.0008a + 0.15568ac^2^- 0.09160	0.984	b	length of head—atlas/axis joint center to end of nose (m)
	flexion/extension	I_yy_	=	-00.00112a - 0.00283a(b^2^+c^2^) + 0.07403	0.720	c	ecto-orbitale breadth (m)
	abduction/adduction	I_zz_	=	-0.00007a - 0.00613a(b^2^+c^2^) + 0.04484	0.708		
Neck	mass	m	=	0.063a + 98.915bd^2^–3.527	0.952	a	Whole body mass (kg)
	internal/external	I_xx_	=	-0.00154a - 0.00313a(e^5^-c^5^)/(e^3^-c^3^) + 0.11749	0.816	b	length from axis (base of the head) to C7 (m)
	flexion/extension	I_yy_	=	-00.0007a + 0.01245a(e/π)^2^+b^2^ + 0.01634	0.871	c	circumference at axis/atlas joint (base of head) (m)
	abduction/adduction	I_zz_	=	-0.00129a + 0.00719a(e/π)^2^+b^2^ + 0.04938	0.976	d	circumference at mid length, halfway between the axis and C7 (m)
						e	circumference at C7/T1 (base of neck/shoulders) (m)
Abdomen	mass	m	=	0.030a + 16.692c(b^2^+bd+d^2^) + 0.106	0.957	a	Whole body mass (kg)
	internal/external	I_xx_	=	0.00814a + 0.00471a(b^5^-d^5^)/(b^3^-d^3^) - 0.28462	0.929	b	circumference at T13/L1 joint (base of ribs) (m)
	flexion/extension	I_yy_	=	00.01457a + 0.01824a(b/π)^2^+c^2^–0.50031	0.653	c	length of T13/L1 to L7/S1 (spinous processes) (m)
	abduction/adduction	I_zz_	=	-0.02726a + 0.00616a(b/π)^2^+c^2^ + 10.08268	0.687	d	circumference at level of waist (narrowest point) (m)
Thorax	mass	m	=	0.094a + 11.135c(d^2^+db+b^2^) + 4.966	0.978	a	Whole body mass (kg)
	internal/external	I_xx_	=	0.00384a + 0.00183a(d^5^-b^5^)/(d^3^-b^3^) - 0.05972	0.935	b	circumference at C7/T1 (base of neck/shoulders) (m)
	flexion/extension	I_yy_	=	-00.00514a + 0.04659a(d/π)^2^+c^2^ + 0.12633	0.739	c	length of C7/T1 joint center to T13/L1 joint center (m)
	abduction/adduction	I_zz_	=	-0.00566a + 0.04428a(d/π)^2^+c^2^ + 0.16146	0.763	d	circumference at T13/L1 joint (base of ribs) (m)
Tail	mass	m	=	0.020a + 22.123bc^2^–0.667	0.934	a	Whole body mass (kg)
	abduction/adduction	I_xx_	=	0.00015a + 0.00198a(c/π)^2^+b^2^–0.01353	0.944	b	length from sacrum to tip of tail (m)
	flexion/extension	I_yy_	=	-0.00042a + 0.00148a(c/π)^2^+b^2^ + 0.00989	0.960	c	circumference at sacrum (m)
	internal/external	I_zz_	=	0.0001a + 0.00173ac^2^–0.00410	0.913		

## Discussion

This investigation offers, for the first time, a complete set of experimental data on the mass and inertial properties of the adult male German Shepherd dog. To date, there has been only one other study that has provided a set such as this, using MRI to indirectly extract body segment data for 3 dogs of mixed breeds [32]. In comparison, by using 6 dogs of the same breed, gender and occupation, our study provides a larger, more homogenous set of data. Moreover, our direct measures of body segment parameters do not need any density assumptions, indeed, we actually determine density as a by-product of our method. The segmentation methods used for current study are similar to those used for the study of mixed breeds except here the tail has been removed and presented as a separate segment and, due to the flexibility of the spine, the trunk has been divided into abdomen and thorax segments so that they may better represent trunk movement.

A comparison of relative mass distribution between the two studies produced similar findings for % body mass and location of CoM. The major difference was in the CoM of the head, which was found to be located 10% more rostral than that of German Shepherds. While the study does not state what breed the dogs were, it was noted that the head of the smallest dog was slightly brachycephalic (flat, wide skull shape) which may have been the source of the variation. Excluding the skull, CoM locations varied between 1.5 and 4.4%, with an average variation of 3.5%. In comparing segment masses normalized to % body mass, again, results were comparable with the greatest variation occurring at the head. The heads of the German Shepherds were found to be an average of 7.7% of body mass compared to an average of 9.2% of body mass for the three mixed breed dogs. Of the remaining segments, corresponding segment masses within the 2 studies varied between 0.08 and 0.39% with an average variation of 0.11%. One other study used CT scans to determine inertial properties for the hind limb of 14 clinically normal Labrador retrievers [31]. Average segment masses were within 0.1% of those recorded here for the pes and crus segments, however the thigh of the Labrador Retriever was found to be 1.5% heavier than that of the German Shepherd. Location of CoM was found to be 4.4, 5.6.and 2.6% more proximal for the pes, crus and thigh, respectively for the Retriever. These differences are relatively small but show potential differences that could be attributed to breed.

The wide variety of morphometric measures used for the regression analyses facilitated segment-specific equations to be determined and strong correlations to be realised. As an example, elbow circumference worked well as a factor for estimating the mass of the brachium segment, however the carpus circumference correlated better for estimating the mass of antebrachium segment. Models for estimating segment mass did not always work for estimating MoI for the same segment, e.g. the neck was best modelled as a cylinder for estimating segment mass, but as a frustum for estimating its MoI.

One problem with using frozen cadavers was the angle of extension in the limbs. Care was taken to ensure each cadaver was sufficiently suspended in stance in the freezer with paws planted. Excessive flexion, for example, could affect underlying bone and tissue position and thereby cause variation in segment mass and circumference. As previously noted [36], MoI is not significantly affected by small errors in mass, however dimensional errors could contribute inaccuracy to the system. A tall dog that has reached the height capacity of the freezer, one who has reached rigor mortise early or any slippage in the suspension mechanism during freezing could introduce such errors. As a result, left and right sided segments were not averaged for each dog but instead were treated as separate segments. Errors in length, in particular the length from the pivot of the pendulum system to the CoM of the box/specimen could also render inaccuracies. To minimize these errors, the length of the pendulum from the frame pivot to the box was measured by a set spacer of known length. The length from the exterior of the box to the CoM of the segment was calculated from 3-dimensional measures taken from the box origin. To avoid possibility of error due to settling, the pendulum length was remeasured after each successive trial. Care was also taken to ensure the assumptions of the simple pendulum was maintained, i.e. the box rotated about the pivot and not from the box/string juncture.

Another potential source for error lay in the dissection of the thigh and brachium, the anatomy of which made it necessary to complete both with compound cuts. The limited abduction range at the shoulder made it difficult to manoeuvre the cut blade in the axial area, while thick muscle mass of the inner thigh and the proximity of the ischium provided limited access to the joint, increasing the opportunity for error.

The greatest benefit to using cadaver segmentation for determining BSPs is that it permits direct measurements of mass, volume, CoM and MoI for individual segments. That being said, as with less invasive means of extracting BSP data, the method has its limitations, the most common being small sample size, as was the case here. Due to a limited supply of sources, most investigations involving cadavers have a small number of specimens and the figures obtained from these populations cannot be used indiscriminately, rather they should be used to examine like groups [18]. It is therefore important that researchers understand how the BSPs were derived and on which populations they may appropriately use them in biomechanical modelling [9, 45].

In comparison to medical imaging, cadaveric segmentation permitted the most direct, cost effective method of measuring moments of inertia, volume and mass, the drawback being that density must be assumed uniform for each segment. Knowledge of the location of various tissue components gained through use of imaging techniques would likely increase the predictive ability of the regression model. For example, a segment may be modelled as a hollow cylinder of bone surrounded by soft tissue, each having a known density.

While freezing the cadaver limits loss of tissue and fluids, it’s important to keep in mind that the inertial properties for each segment are only accurate for the position in which it was frozen. Changes in muscle diameter and position through movement would cause variations in the inertial properties, however since a dynamic MoI measure is not yet possible, the static measure is an acceptable representation.

Despite the small sample size, good correlations were found for the inertial properties of the segments, and these data provide a full set of biomechanical segment parameters for the German Shepherd dog. With caution, they may also be applied to other dog breeds of similar stature, such as the Golden Retriever or Malinois or in the absence of breed-specific data. In a breed-specific study comparing Greyhounds to Labrador Retrievers, kinematic patterns greatly differed, likely due to differences in pelvic mechanics [18]. It is also important to note that when performing an objective gait analysis on dogs, for quantitative gait analysis to be comparable between dogs, within dogs, between sides (i.e. left and right) and between fore and hind limbs, they must be traveling at a constant velocity in a symmetrical gait such as the trot [46–48]. Even within the trot there is some speed-related variation in kinematic pattern [16] therefore it’s possible that a treadmill-based design may afford greater control over the stride velocity than that of over-ground trotting. Future studies are needed to determine whether mass distribution over individual segments is consistent between dogs of different breeds and sizes. It is hoped that in time, the movement patterns produced by gait model may reflect compensatory behaviour that would otherwise be difficult to interpret to the untrained observer. In summary, an objective gait analysis including detailed joint mechanics could augment as a clinical diagnostic tool for identifying pathologies associated with gait anomalies such as this, impacting future evaluations of musculoskeletal disorders and therapeutics [15].

## Supporting information

S1 File Canine informed consent. (DOC)
