# Supplementary material for: Inertial properties of the German Shepherd Dog
Source: PLoS One. 2018 Oct 19;13(10):e0206037. doi: 10.1371/journal.pone.0206037 (PMC6195294; doi:10.1371/journal.pone.0206037)
Supplement: S1 File — (DOC) [file pone.0206037.s001.doc]

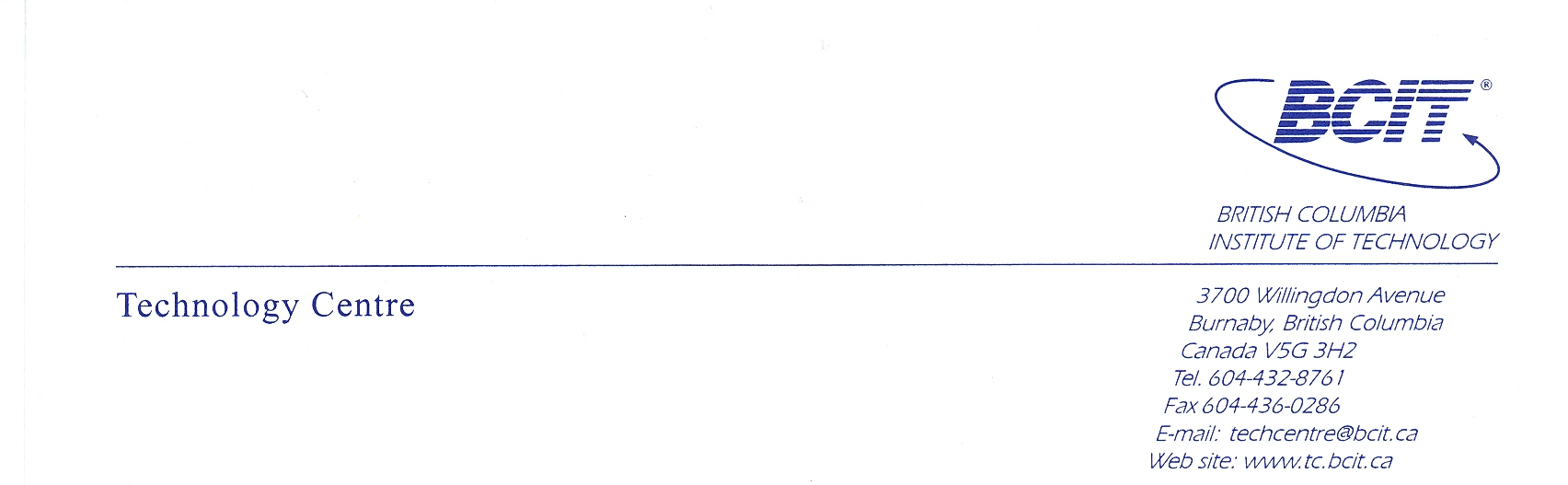


1.Nov.2006

## RESEARCH CONSENT FORM

**Development of a Link Segment (Mathematical) Model**

**of the German Shepherd Police Dog**

**Principal Investigator*:***

Dr. Silvia Ursula Raschke, Technology Centre, (604) 412-7597

**Purpose:**

The purpose of this project is to build a mathematical model of an ‘average’ police dog using measurements taken from the bodies of a minimum of 5 (five) police dogs. This model will be a key foundational element in the development of a Canine Research Centre focusing on police dog performance and health in the BC Lower Mainland. Link segment models are essential in all research measuring the movement and gait of dogs. This will be particularly important after injury and during rehabilitation as it will then be possible to measure changes that can not be assessed by the eye alone.

**Study Procedures:**

How will the model be developed?

- The euthanized body will carefully be dissected into locomotor (movement) segments.

*(Eg: paw, lower leg, thigh and pelvis, for the hind limb).*

- Using special tools, each segment will be measured for weight, length, circumferences, volume and the location of the centre of mass.
- The measurements will be collected in a spread sheet and averaged across dogs
- Finally, the resulting data will be used to develop the model of an average German Shepherd police dog.

**We will be able to begin carrying out dissections as of December 1, 2006.**

What happens after the dissection is completed?

After dissection, the dog’s remains will be taken to a crematorium for cremation and the dog's ashes will be returned to you.

The researchers will, at all times, treat your dog’s body with respect.

**Eligibility*:***

Eligible dogs must be:

- Over 2 (two) years of age
- Both active duty and retired dogs
- German Shepherd Police Dogs that have died of natural causes, illness, minor trauma or most diseases, or have had to have been euthanized for purposes other than this study.

**Exclusions*:**

Which dogs are not eligible?

- Dogs under 2 (two) years of age
- Dogs with diseases or conditions that are communicable to other dogs or humans
- Dogs that have had amputations
- Dogs that are being euthanized due to major traumatic injuries

*Note: As the protocol is developed it may be necessary to add to the exclusion list. Should there be any changes the veterinarian listed by you will be informed so that the information can be added to the dog’s records.

**Risks:**

There are no known risks to this study.

# Benefits:

Donating your dog’s body to the project will allow the first ever mathematical model of the German Shepherd to be developed. This model will be the foundational element of two streams of research proposed at the K9 Centre for Performance and Health. **Both these streams of research will help all future dog handlers and their dogs lead safer and healthier lives, in addition to improving performance on the job.** They are:

1. Rehabilitation research will use the model to set standards of practise for the rehabilitation of injured police dogs. Just as Olympic athletes benefit from specialised research into athletic performance, police dogs will be able to benefit from research into their specific performance and medical needs. This in turn will benefit the average pet dog, just as sports medicine research has benefited the ordinary weekend athlete.
2. Performance research will use the model to focus on training and handling methods. This research stream will be police service driven and will involve the evaluation of various training and handling methods to determine which produce measurable results and which don’t. Research goals in this area focus on reduction of risk of injury and to better quantify the contribution these dogs make to the community. The results will be incorporated into the professional environment at both the VPD and the RCMP.

**Confidentiality:**

Information on individual dogs and their handlers will be kept strictly confidential. Copies of this consent form will be kept in a locked cabinet that is only accessible to researchers at the BCIT Technology Centre. It will be the only document identifying you and your dog. Neither you nor your dog will be identified by name in the database or in any reports on the completed study. Your dog will only be identified by a coded number.

**Remuneration/Compensation:**

Donating your dog to the study will not cost you anything. On euthanization, you will leave your dog’s body with your veterinarian, where it will be picked up either by a BCIT researcher or by a representative of the RCMP. The costs of cremation will be covered by the research funding, as will the cost of returning your dog’s ashes to you in the container provided by the crematorium.

If you have any questions or desire further information with respect to this study you should contact Dr. Silvia Raschke at (604) 412-7597.

**New Findings:**

If you choose to register your dog for this study you will be advised of any new information that may affect your willingness to make that donation at the time that your dog passes away, as soon as that information is available to researchers.

**Note: Detach this page and return in self addressed envelope supplied. Keep remainder of document for your files.**

**Subject Consent:**

I understand that my donation my dog’s remains to be used in this study is entirely voluntary on my part and that I may change my decision to make that donation at any time in the future or at the time of the dog’s death, without any consequences. I have received a copy of this consent form for my own records.

I wish to donate me dog’s remains to this study, at the time of his or her death.

Owner Signature Date __________

Witness Signature Date___________

Investigator’s Signature Date

**Dog’s Name: _______________________**

**Dog’s Age: _______________________**

**Dog’s Sex: male / female (circle one)**

| **Handler’s Contact Information:** | **Veterinary Contact Information:** |
| --- | --- |
| Name: ______________________  Preferred Mailing Address:  ____________________________  ____________________________  ____________________________  ____________________________  Preferred Telephone: ___________  e-mail: _______________________ | Name: ______________________  Clinic Name:_________________  Mailing Address:  ____________________________  ____________________________  ____________________________  ____________________________  Telephone:___________________  Fax: :________________________  e-mail: _______________________ |
